# Designing a Trauma-Informed Safety by Design Framework for Nonconsensual Intimate Image Removal Platforms: A 2-Phase Sequential Exploratory Study

**DOI:** 10.2196/94438

**Published:** 2026-08-10

**Authors:** Ji-yeon Lee, Yejun Koh

**Affiliations:** 1 Department of Counseling-UX Psychology, Hankuk University of Foreign Studies, 107 Imun-ro, Seoul, 02450, Republic of Korea, 82-2-2173-3086.

**Keywords:** nonconsensual intimate images, persona, Safety by Design, trauma-informed design, trauma, online safety, consent

## Abstract

**Background:**

Removal platforms for nonconsensual intimate images (NCIIs) are essential, as they have become the recourse for victims of such abuse; yet their interfaces are not designed around the cognitive and emotional realities of trauma. Safety by Design directs platforms to protect vulnerable users but not how; trauma-informed care defines what that protection requires, while usability heuristics keep interfaces usable but are not themselves trauma-sensitive. However, no framework integrates them into design guidance that is both usable and protective for NCII removal platforms.

**Objective:**

This study develops trauma-informed design, a cohesive framework that integrates trauma-informed care with established usability heuristics under Safety by Design, so that NCII removal platforms protect trauma-affected users while remaining realistically usable; a preliminary, domain-informed evaluation provides initial supporting evidence.

**Methods:**

We used a 2-phase sequential exploratory design, drawing on a globally accessible NCII removal platform as an illustrative case. Phase 1 developed trauma-differentiated personas and mapped each persona’s user journey to surface pain points that varied by level of trauma. Phase 2 analyzed a focus group of 8 female Counseling-UX (User Experience) Psychology students who had completed coursework in user psychological safety with consensual qualitative research methodology. Participants assessed the plausibility of the personas and the proposed framework with respect to perceived emotional safety and perceived usability.

**Results:**

Mapping each persona’s journey surfaced pain points that differed by level of trauma, which were synthesized into a 6-guideline trauma-informed design framework: emotional scaffolding, process transparency, self-pacing mechanisms, institutional trust signals, context-aware support, and grounding tools. In phase 2, domain-informed evaluators judged the personas plausible, and the framework directions emotionally safer, and potentially more usable.

**Conclusions:**

Integrating trauma principles into user experience evaluation can help identify and address psychological barriers in NCII removal platforms. The framework provides the preliminary, domain-informed foundation that trauma-sensitive design of such platforms requires. Because the framework was not tested with NCII victim-survivors or users actively seeking image-removal support, findings represent preliminary plausibility and acceptability evidence rather than ecological validation or real-world effectiveness.

## Introduction

### Background

Nonconsensual intimate image (NCII) removal platforms are an essential recourse for victims of NCII abuse. Yet despite real advances in content hashing and proactive detection [1], most removal workflows were built around takedown logistics rather than the psychological realities of trauma, leaving help-seeking more burdensome and distressing than it needs to be [2,3]. Because users must navigate these interfaces while in acute distress, the burden falls on the very people the system aims to protect, thereby contravening Safety by Design’s principle that safety should never rest solely on the user [4]. These concerns can deter victim-survivors from seeking help altogether [5]. The problem is therefore not merely technical or regulatory but clinical: it requires understanding how trauma reshapes the cognitive and emotional capacities that digital interaction demands.

Empirical accounts of how victim-survivors experience reporting and removal systems reinforce this concern. Studies of technology-facilitated abuse document that survivors frequently encounter fragmented, effortful, and opaque help-seeking pathways, and that maintaining safety online imposes a substantial and sustained burden [3]. Research on image-based sexual abuse describes the compounding distress victim-survivors face when navigating institutional and platform responses that are difficult to access, slow, or inadequately explained [6]. Controlled comparison of NCII reporting interfaces across 45 platforms shows that the interaction style of the report itself produces measurable trade-offs in clarity, efficiency, and elicited distress [7]. Survivor-centered work developing digital tools for gender-based violence has further shown that survivor-facing systems often fail not because the underlying technology is inadequate but because they are not designed around victim-survivors’ psychological and contextual realities [2]. Taken together, this evidence indicates that the limitations of current NCII removal platforms are experienced by users at the point of help-seeking and justify a design approach grounded explicitly in trauma psychology.

Safety by Design [4], trauma-informed care (TIC) [8], and usability heuristics [9] each address part of what NCII removal platforms require, but none integrates the others into interaction-level guidance, a gap this work addresses. Recent NCII-specific scholarship has mapped how harms arise across sociotechnical layers and where interventions might sit [10], yet no existing work integrates clinical trauma theory, usability evaluation, and platform-level safety engineering into an actionable implementation framework for NCII removal platforms.

The insufficiency of any single tradition is clearest at the points where NCII removal platforms can fall short for the users they aim to serve: the gaps tend to surface at the interaction level, when a victim-survivor must initiate and sustain a multistep reporting process while managing acute distress. Removal workflows implicitly assume a trauma-free user who can tolerate ambiguity, accept opaque data handling, and execute irreversible actions under stress. In contrast, the population served is better characterized by reduced perceived control, dissociative disengagement when retriggered, and fragile reengagement after interruption [11,12]. Such states may manifest in predictable ways: dissociation may interrupt a multistep process, hyperarousal may prompt premature abandonment, shame may inhibit the disclosure some steps require, and learned helplessness may impede reengagement after a process breaks [12]. These patterns are consistent with the clinical conditions that characterize this population, grounded in the neurobiology of interpersonal trauma [13]: the interface tends to assume a user who can negotiate such moments, whereas the trauma-affected user’s more likely response may be to withdraw.

To address this gap, we develop and preliminarily evaluate a trauma-informed design (TID) framework for NCII removal platforms through a 2-phase sequential exploratory design. The aim is not to evaluate any single service but to provide a detailed framework, grounded in Safety by Design, for supporting victim-survivors of NCII abuse across removal platforms. The term “trauma-informed design” is not new: related approaches have been advanced for digital systems, including trauma-informed computing [1] and TID heuristics for a social service website [14], building on TIC [8]. Our contribution is not the term but its operationalization, which provides concrete, evaluable design guidance for NCII removal platforms, grounded in Safety by Design. Specifically, we apply trauma-differentiated personas to a representative NCII removal platform to identify barriers invisible to any single framework and subsequently assess candidate design responses with domain-informed student evaluators. By integrating clinical trauma knowledge into user experience (UX) evaluation, this study addresses design barriers shaped by trauma-altered cognition, affect, and behavior among NCII removal platform users. The contributions are 3-fold: (1) a diagnostic methodology bridging TIC, Safety by Design, and usability evaluation to identify psychological design gaps invisible to any one approach; (2) 6 preliminary TID guidelines; and (3) preliminary evidence that domain-informed student evaluators perceived the personas and several design responses as plausible, emotionally safer, and potentially more usable.

### Research Questions

The research questions (RQs) are as follows:

- RQ1. When trauma-differentiated personas are applied to the user journey, what trauma-related barriers emerge that conventional usability heuristics alone may not fully capture?
- RQ2. Under Safety by Design, how can TIC and usability heuristics principles be integrated into design guidance that addresses these barriers?
- RQ3. How do domain-informed evaluators judge the plausibility of the trauma-differentiated personas and the resulting design directions with respect to perceived emotional safety and perceived usability?

## Methods

### Conceptual Framework

Three established traditions each address part of this problem at a different level, and the present framework integrates them. Safety by Design sets the overarching mandate: platforms, not users, must bear the burden of protection (the why); TIC supplies the clinical content of that protection (the what); and usability heuristics govern how the interaction is delivered (the how). We take each in turn and then identify the gap that remains.

### Safety by Design

Safety by Design embeds user safety into digital products from conception rather than retrofitting protections after harm [4,15]. Australia’s eSafety Commissioner established the framework around 3 overarching principles: Service Provider Responsibility (safety burdens never fall solely on users), User Empowerment (protective defaults with meaningful user controls), and Transparency and Accountability (published enforcement data and accessible policies). The Trust and Safety Professional Association [16] operationalizes these through implementation guidance including proactive risk assessment, friction features (interstitials, pause points, and content warnings), and holistic safety across the full product life cycle.

### TIC: Clinical Foundations

TIC originated in behavioral health as an organizational framework that reframes the service encounter from “What is wrong with you?” to “What happened to you?” [8,15]. Following the Substance Abuse and Mental Health Services Administration framework [8], trauma-informed practice is organized around 6 principles: Safety; Trustworthiness and Transparency; Peer Support; Collaboration and Mutuality; Empowerment, Voice, and Choice; and Cultural, Historical, and Gender Issues. These principles, rather than recovery-oriented treatment, anchor and are operationalized by the present framework. The epidemiological basis is substantial: approximately 90% of US adults have experienced at least 1 traumatic event [17], and World Health Organization data indicate 70.4% lifetime trauma exposure across 24 countries [18]. Adverse childhood experiences alone generate an estimated US $581 billion in annual costs across Europe and North America [19].

For digital platforms serving NCII victim-survivors, the assumption of a nontraumatized user is empirically untenable [1]. This is where TIC’s clinical specificity becomes essential for design: trauma does not merely cause distress; it structurally alters the cognitive and emotional architecture that digital interaction presupposes. Posttraumatic stress disorder (PTSD) disrupts executive functions including planning, sustained attention, and working memory [11], precisely the capacities that complex multistep digital workflows demand. Trauma erodes institutional trust [12], making conventional credibility signals insufficient. It produces hypervigilance toward perceived threats, meaning that ambiguous interface elements are processed as dangerous rather than neutral. These are not usability problems in the conventional sense; they are clinical phenomena requiring clinical understanding. TIC’s Physical Environment domain provides the conceptual bridge: if physical environments must embody TIC through architecture and spatial design, digital environments must do the same through visual design, information architecture, and interaction flows.

A consistent principle across the sexual violence recovery literature is that disempowerment and loss of control are central to the injury, and that restoring a victim-survivor’s sense of control and agency is therefore central to recovery [12,20-22]. This reframes perceived control as more than a clinical outcome: it is a design-relevant construct. Interactions that compel a survivor to surrender control (eg, opaque eligibility decisions, irreversible actions, and unexplained delays) risk reenacting the disempowerment of the original harm, whereas interfaces that return choice, pacing, and transparency can reinforce the recovery process itself. TID accordingly treats the restoration of control and agency as the through line linking clinical trauma care to interaction design, and the design guidelines developed here can be read as its concrete instantiations.

The convergence with TIC is significant: Service Provider Responsibility, User Empowerment, and Transparency parallel TIC’s institutional accountability, its Empowerment, Voice, and Choice principle, and Trustworthiness. The 2 operate at different levels; however, Safety by Design governs how platforms and policies are structured, whereas TIC governs how interactions feel and what they presuppose about the user’s psychological state. NCII removal platform design therefore requires both structural safety and psychological specificity.

### Usability Heuristics: Convergence With and Limits Beyond TIC

Usability heuristics map onto TIC with notable directness [9]: system status visibility corresponds to Trustworthiness, user control to Empowerment, consistency to Safety through predictability, and recognition over recall to Safety through cognitive load reduction [14]. This convergence suggests that good usability and TID share common ground: both prioritize clarity, control, and predictability. However, the convergence has critical limits. Heuristics assess whether interfaces are learnable and efficient for a general user population. They cannot assess whether an interface is emotionally safe, whether it accommodates the specific cognitive deficits produced by PTSD, or whether it replicates relational dynamics of powerlessness (eg, forced workflows without exit, absence of opt-out mechanisms, and institutional opacity that mirrors abuser control) [23]. For NCII removal platforms where users may be in acute crisis, cognitive load reduction is not an enhancement but a prerequisite. This gap between what heuristics detect and what trauma-informed evaluation requires defines the conceptual space TID occupies.

### The Remaining Gap

**Figure 1.**
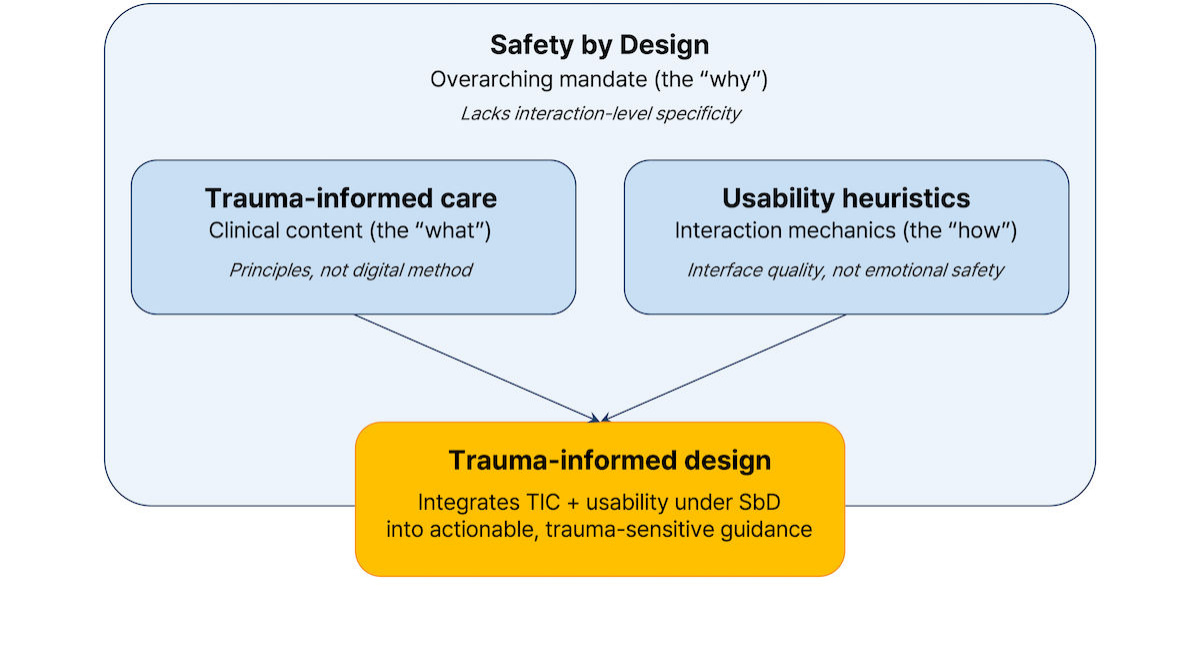
Conceptual framework for trauma-informed design integrating trauma-informed care, Safety by Design, and usability heuristics. SbD: Safety by Design; TIC: trauma-informed care.

Trauma-informed computing maps trauma principles onto design but warns that design research can resemble psychotherapy without safeguards [1]; like a subsequent scoping review [5], it remains at the principle-mapping stage. Design-justice work further shows that safety cannot be imposed through expert assumption alone but must be grounded in victim-survivors’ lived realities [2]. The gap is thus 3-fold: TIC supplies principles without a digital methodology, usability heuristics supply evaluation tools without emotional safety assessment, and Safety by Design supplies structural mandates without psychological specificity. To date, no existing work integrates all 3 into an operationalized framework for NCII removal platforms. Figure 1 illustrates the 3 traditions, their limits, and how TID bridges them.

### Research Design

**Figure 2.**
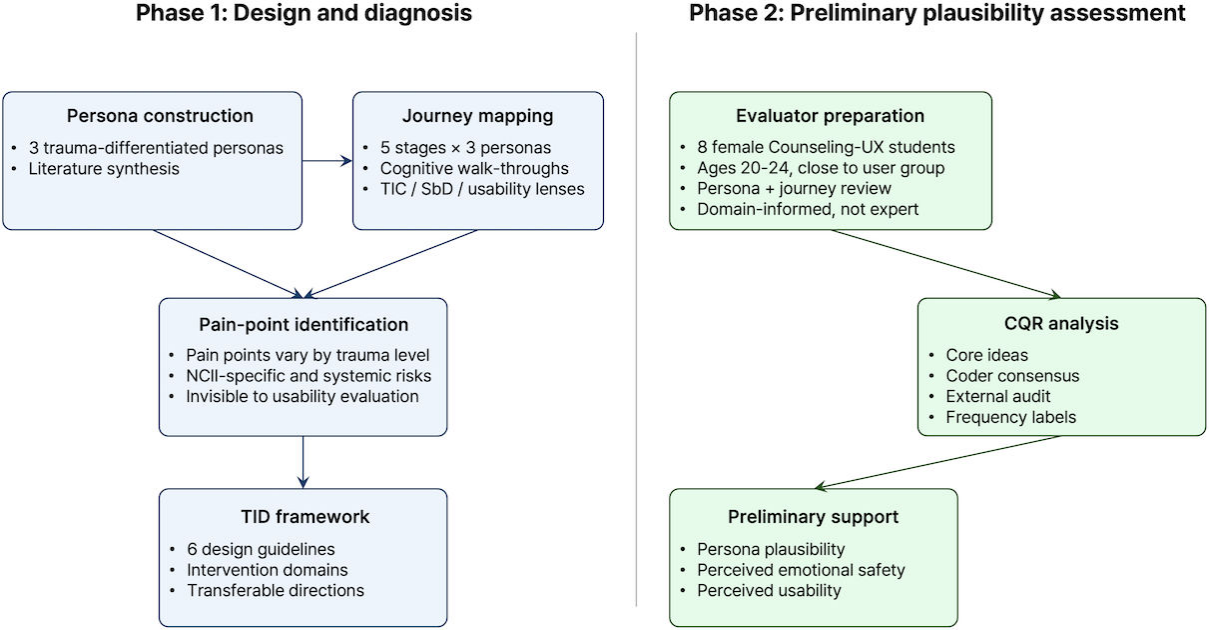
Research design overview. CQR: consensual qualitative research; NCII: nonconsensual intimate image; SbD: Safety by Design; TID: trauma-informed design; UX: user experience.

This study used a 2-phase sequential exploratory design [24]. Phase 1 (Design and Diagnosis) constructed trauma-differentiated personas and applied them via User Journey Mapping to identify TIC and Safety by Design misalignments in an existing NCII removal platform. Phase 2 (Domain-Informed Evaluation) evaluated persona plausibility, perceived emotional safety, and perceived usability of TID-informed design response materials through consensual qualitative research (CQR) [25]. The study was conducted at Hankuk University of Foreign Studies in Seoul, South Korea, from March to August 2025, as a preliminary framework development and plausibility evaluation study. Figure 2 illustrates the overall design.

A globally accessible NCII removal platform was selected as the case based on its content-hashing technology, international user base, and public accessibility [26]. Platform-identifying details are anonymized to protect ongoing operations.

### Persona Methodology and User Journey Mapping

The persona methodology [27] creates fictional yet research-grounded user profiles to anchor design decisions in human needs rather than abstract specifications. Personas range along a continuum from assumption-based to data-driven [28]. Our personas were constructed from an evidence synthesis of published findings on NCII victimization, trauma psychology, and adolescent digital risk, rather than from direct survivor interviews, reflecting a deliberate ethical decision to avoid premature survivor involvement in an early-stage framework study [1,29]. Acknowledging the stereotyping risks inherent in personas constructed without direct user participation [28,30], we used 2 mitigations: grounding every persona characteristic in published empirical findings and subjecting personas to preliminary plausibility assessment through CQR with domain-informed student evaluators.

User journey mapping [31] visualizes emotional, cognitive, and behavioral arcs across interaction touchpoints. We adapted it from a generative design tool into a diagnostic instrument: rather than designing new interactions, we used trauma-differentiated personas to surface where existing interactions violate TIC, Safety by Design, and usability principles. This constitutes a specialized trauma-informed variant of the product risk assessments recommended in Safety by Design practice [16], enriched by clinical understanding of how trauma shapes each journey stage.

### Phase 1: Persona Construction and Journey Mapping

The personas were developed by the authors through an iterative, evidence-grounded process. Candidate user vulnerabilities, help-seeking contexts, and platform interaction risks were generated and checked against evidence on NCII victimization [1,6], adolescent digital risk [32,33], and intimate partner violence [3,23]. Dimensions that recurred across these sources (trauma severity, perceived control, institutional trust, developmental status, help-seeking autonomy, and capacity to sustain a multistep digital task) differentiated the personas; perceived control was treated as central because it predicts help-seeking engagement and tolerance for uncertainty [34]. The personas were reviewed against Herman’s [12] trauma continuum to ensure clinically meaningful rather than merely demographic differentiation, yielding 3 profiles presented in the Results section (Table 1): Sara (low-moderate trauma), Rina (severe trauma with complex PTSD indicators), and Minji (adolescent acute distress with developmental vulnerability).

**Table 1.** Trauma-differentiated personas.

Dimension	Sara	Rina	Minji
Age (sex)	28 years (female)	32 years (female)	16 years (female)
Context	Ex-partner shared images postbreakup	Abusive partner distributed images during prolonged IPV^a^	Peer coercion; fear of image leak
Trauma level	Low-moderate	Severe (complex PTSD^b^ indicators)	Acute distress and developmental vulnerability
Key drivers	Distrust; desire for clarity	Shame, hypervigilance, and learned helplessness	Fear, confusion, and isolation
Primary goal	Remove images via transparent process	Regain control; requires emotional safety	Understand eligibility; age-appropriate support
TIC^c^ principles	Trustworthiness and empowerment	Safety, empowerment, and collaboration	Trustworthiness, safety, and empowerment
Safety by Design	Transparency and provider responsibility	User empowerment and friction features	Provider responsibility and age-specific risk

a IPV: intimate partner violence.

b PTSD: posttraumatic stress disorder.

c TIC: trauma-informed care.

Each persona was applied to 5 interaction stages (Awareness, Consideration, Decision, Action, and Postaction) [31], using a structured matrix documenting anticipated needs, actions, barriers, proposed design responses, and the TIC, Safety by Design, and usability principles implicated. At each stage, the authors recorded both what a standard usability evaluation (Nielsen’s heuristics) would flag and what clinical trauma knowledge (TIC and Safety by Design) would flag, surfacing features that can pass conventional heuristic evaluation while failing trauma-informed evaluation. The authors completed the matrix independently and reconciled differences through structured discussion; claims that could not be reconciled were narrowed or excluded. The resulting journey maps appear in the Results section (Table 2).

**Table 2.** Trauma-differentiated user journey maps.

Persona and stage	Needs	Action	Current barrier	TID^a^-informed response	TIC^b^	Safety by design
Sara (low-moderate)						
Awareness	Find credible platform	Searches removal	Difficult to locate via search	Visible entry points; onboarding	Trustworthiness	Transparency
Consideration	Understand function	Reads home page cautiously	Governance and data handling unclear	Plain-language data explanations	Trustworthiness	Transparency
Decision	Reassurance; error protection	Attempts case creation	No preview; fears irreversibility	Gentle prompts; preview before submission	Empowerment	Safety
Action	Privacy reassurance	Uploads content hash	Upload triggers shame; no support	Calming messages; grounding tools	Safety	Protective defaults
Postaction	Affirmation; status visibility	Waits for confirmation	Insufficient status visibility	Affirming follow-up; status tracking	Trustworthiness	Accountability
Rina (severe)						
Awareness	Welcoming language	Hesitant search; delays	No welcoming or survivor voice	Affirming, survivor-centered entry	Safety, empowerment	Proactive
Consideration	Non-triggering content	Briefly visits platform	Elements not validated as safe	Validated non-triggering UI^c^	Safety	Protective defaults
Decision	Control; ability to pause	Attempts case but leaves	Rigid; no save; no support	Grounding techniques; save draft	Empowerment	Friction features
Action	Pause without losing progress	Reattempts upload	No support; solitary completion	Pacing indicators; helplines	Safety, collaboration	Empowerment
Postaction	Long-term support pathways	Seeks external support	No pathways to long-term support	Connect to NGOs^d^, peer networks	Collaboration	Holistic
Minji (adolescent)						
Awareness	Age-appropriate guidance	Searches due to fear	Unclear guidance for minors	Messaging for minors; youth links	Trustworthiness	Age-specific risk
Consideration	Verify eligibility	Reads cautiously	FAQ–UI^c^ conflict	Simplified FAQ^e^; consistent messaging	Trustworthiness	Transparency
Decision	Clarity on access	Looks for eligibility	No minor-specific jurisdiction info	Age-specific legal guidance	Empowerment	Provider responsibility
Action	Support if restricted	Attempts case creation	Block; no guidance or support	Clear alternative steps; reassurance	Collaboration	Empowerment
Postaction	Next-step guidance	Redirected to generic list	Generic list; no minor help	Visible helplines; tailored follow-up	Empowerment	Proactive

a TID: trauma-informed design.

b TIC: trauma-informed care.

c UI: user interface.

d NGOs: nongovernmental organizations.

e FAQ: frequently asked questions.

Journey findings were translated into low-fidelity TID-informed interface examples for use as phase 2 evaluation stimuli, each reviewed through scenario-based cognitive walk-throughs [35]; these examples were not treated as final design solutions. Crosscutting barrier themes were then derived by grouping recurrent persona-stage barriers and cross-walking each against the TIC principle, Safety by Design principle, and usability heuristic it implicated. The 6 TID guidelines were synthesized from these themes by consolidating barriers with shared design implications into broader intervention targets (eg, opaque data handling, unclear steps, and uncertain timelines into process transparency). Each of the 5 crosscutting barriers was mapped to 1 or more guidelines (Table 3), and every guideline was anchored to the TIC principle and Safety by Design mandate it operationalizes; guideline derivation and labeling followed the same independent-coding-then-consensus procedure used for journey mapping.

**Table 3.** TID^a^ guideline framework: derivation and evaluated design responses^b^.

Guideline (domain)	Barrier addressed	Persona(s) most affected	TIC^c^	Safety by design	Usability-heuristic overlap	Design response	Interpretive status (phase 2)
G1: Emotional scaffolding (visual and emotional tone)	Emotionally triggering content	Rina, and Sara	Safety	Service-provider responsibility	Aesthetic and minimalist design	Reduce threat cues; supportive language	Perceived as more comforting
G2: Process transparency (language and framing)	Opaque data handling; ambiguous eligibility language	Sara, and Rina	Trustworthiness and transparency	Transparency	System status visibility	Make process, data use, and timelines explicit	Perceived as trust-supporting
G3: Self-pacing mechanisms (self-pacing and grounding)	Multi-step workflows without exit	All	Empowerment, voice, and choice	User empowerment	User control; help documentation	Enable pause, save, and return without penalty	Perceived as reducing pressure
G4: Institutional trust signals (information architecture)	Low institutional credibility	All	Trustworthiness and transparency	Service-provider responsibility	(No direct equivalent)	Show recognizable affiliations and security cues	Consistently perceived as trust-supporting
G5:Context-aware support (information architecture)	Absent tailored or contextual support	Minji, and Sara	Cultural, historical, and gender issues	Service-provider responsibility	Consistency; system–-world match	Tailor guidance to age, jurisdiction, and need	Identified as relevant; requires survivor-centered testing
G6: Grounding tools (self-pacing and grounding)	Absent emotional support	All	Peer support	User empowerment	User control; help documentation	Provide grounding and immediate support options	Perceived as usable; not behavioral evidence

a TID: trauma-informed design.

b G1-G6 denote the 6 TID guidelines. Columns map each guideline to the barrier it addresses, the personas most affected, its TIC principle and Safety by Design mandate, and the overlapping usability heuristic [9]; the final 2 columns give the candidate design response and its phase 2 interpretive status. Design responses were evaluated as plausibility and acceptability stimuli by domain-informed student evaluators, not tested behaviorally with nonconsensual intimate image victim-survivors or active help seekers.

c TIC: trauma-informed care.

### Phase 2: Domain-Informed Plausibility Evaluation

Eight female university students (aged 20‐24 years) majoring in Counseling-UX Psychology at a Korean university were recruited through purposive sampling as domain-informed student evaluators. Participants were selected for their uncommon dual training in counseling psychology and UX: few individuals, and particularly established specialists, command both, yet this combination is what allows an evaluator to read an interface through both the usability-heuristics and the TIC lenses the framework integrates. As young women in the age range most affected by NCII and image-based sexual abuse, they were also demographically closer to the affected user population than older specialists would be, which supported empathic appraisal of the personas’ experiences. They were not recruited as credentialed experts, NCII survivors, representative end users, or individuals actively seeking image-removal support. Because trauma-informed digital safety design is an emerging interdisciplinary area and formally established specialist roles are limited in the Korean context, this group was used for preliminary plausibility assessment rather than expert validation. The 8 evaluators were distinct from the persona development team, preserving the independence of the appraisal. Prior to the focus group, participants received a structured briefing on the study purpose, the NCII removal platform context, the 3 persona summaries, a brief explanation of the TID framework and its 6 guidelines, and the distinction between evaluating plausibility and reporting personal trauma experience. The 1-hour semistructured focus group was conducted in a private university setting by a researcher trained in Counseling-UX Psychology. The focus group guide covered four blocks: (1) initial platform navigation and spontaneous reactions; (2) evaluation of persona and journey plausibility; (3) side-by-side comparison of the original interface and TID-informed interface examples instantiating the 6 guidelines, including whether the design responses cohere with the framework; and (4) reflection on emotional safety and willingness to use. The focus group guide is provided in Multimedia Appendix 1. Audio was transcribed verbatim in Korean with representative quotes translated into English by a bilingual researcher and verified by a second.

CQR was selected for its emphasis on researcher consensus through team-based analysis and its demonstrated suitability for exploratory research with small, homogeneous samples [25,36]. Analysis proceeded through 5 steps. First, the 2 coders independently reviewed the transcript and segmented participant responses into meaning units. The 2 coders were a master’s-level practitioner in counseling psychology and the first author (JL), a licensed counseling psychologist with expertise in trauma, digital mental health, and human-computer interaction; assumptions and potential biases were documented prior to analysis following CQR protocol [25] and trauma-informed research guidelines [1]. Second, each analyst abstracted the essential meaning of each excerpt into preliminary core ideas, preserving the participant’s intended meaning. Third, the analysis team met to compare core ideas, discuss discrepancies, and reach consensus through iterative discussion. Disagreements were resolved by returning to the transcript and evaluating which interpretation was most strongly supported by the raw data. Fourth, agreed-upon core ideas were clustered into thematic domains and categories through cross-analysis across participants. Fifth, an external auditor, who is a counseling psychology faculty member specializing in digital mental health and was not involved in data collection, reviewed the thematic domain structure, category labels, and representative excerpts, in line with established qualitative trustworthiness criteria [37]. Auditor feedback was discussed by the analysis team, and revisions were made when the audit identified overinterpretation, unclear category boundaries, or insufficient evidentiary support. Frequency labels followed CQR conventions: general (7‐8 of 8 participants), typical (4-6), and variant (2-3).

### Ethical Considerations

This study was reviewed and approved by the Institutional Review Board of Hankuk University of Foreign Studies (approval number HUFS-2507‐009). It was conducted as a preliminary, minimal-risk UX and plausibility evaluation with adult student evaluators; participants were not recruited as NCII victim-survivors, were not asked to disclose personal NCII victimization or trauma histories, and evaluated summarized persona, journey map, and interface-example materials rather than reporting personal experience. Written informed consent was obtained before participation; participants were advised in advance that the evaluation materials addressed NCII and could be distressing, that they could decline any question or pause or withdraw at any time without penalty, and that psychological support was available, if needed. Privacy and confidentiality were protected by deidentifying transcripts, reporting summarized findings rather than identifiable raw statements, and limiting data access to the research team. Safeguards included advance briefing, unrestricted withdrawal rights, on-site counselor availability during and after the session, and summarized rather than raw presentation of potentially triggering NCII content [1]. No compensation was provided.

## Results

### Phase 1: Personas, Journey Maps, and Barrier Identification

Phase 1 produced 3 trauma-differentiated personas, summarized in Table 1. Each persona encodes a distinct configuration of trauma severity, perceived control, institutional trust, and capacity to sustain a multistep digital task under stress. Applying each persona across the 5 interaction stages produced the journey maps presented in Table 2, which document, for each stage, anticipated needs, likely actions, the current barrier, a TID-informed response, and the corresponding TIC and Safety by Design principles.

For Sara (low-moderate trauma), absent procedural clarity and opaque data governance violated Trustworthiness and Safety by Design’s Transparency principle [23,38,39]. Although a general population user might tolerate ambiguous data-handling information as a minor frustration, Sara’s trauma-eroded institutional trust transforms opacity from a usability irritation into a barrier to engagement. Specifically, she cannot proceed without understanding exactly how her sensitive data will be handled, by whom, and under what legal protections. For Rina (severe trauma), the platform’s rigid, linear workflow, which lacked any save-progress functionality, forced a complete restart after trigger-induced abandonment. Given that PTSD specifically disrupts executive functions including planning, sustained attention, and working memory [11], this rigid workflow created a functionally inaccessible environment for users in exactly the crisis state the platform was designed to serve. The misalignment was dual: TIC’s Empowerment, Voice, and Choice principle requires that users maintain control over the pace and direction of their engagement, while Safety by Design’s friction feature guidance recommends pause points and save states precisely to prevent forced completion designs [16]. For Minji (adolescent), ambiguous eligibility criteria violated Trustworthiness: a minor navigating a confusing legal landscape needs unambiguous guidance, not contradictory information across frequently asked questions and interface elements. Generic referral on restriction (ie, redirecting a distressed minor to an undifferentiated list of organizations without age-specific guidance) violated both Safety and Safety by Design’s age-specific risk assessment requirement [40,41].

Five crosscutting barrier themes were synthesized across all 3 journey maps (Table 3): ambiguous eligibility language, emotionally triggering content, absent emotional support mechanisms, opaque data handling, and low institutional credibility. Each theme represents a convergent pattern across clinical, platform-safety, and usability lenses rather than a single-source observation; the full derivation appears in Table 3.

### Proposed TID Framework

The 6-guideline TID framework (Table 3) integrates TIC and Safety by Design principles across 4 intervention domains: Visual and Emotional Tone, Language and Framing, Information Architecture, and Self-Pacing and Grounding. The framework is specified at the level of design principles and the psychological targets they address; the concrete interface features named under each guideline are illustrative instantiations used here as evaluation stimuli, rather than prescribed or finalized designs for any particular service.

Guideline 1 (Emotional Scaffolding): calming color palettes, nontriggering imagery, and empathetic language implemented as platform defaults rather than user-selected options. This guideline targets trauma-related hyperarousal and the appraisal of otherwise neutral interface cues as threatening [12,13]. Guideline 2 (process transparency): plain-language explanations of data handling, process steps, and expected timelines at every interaction stage. It addresses the hypervigilant threat monitoring associated with eroded institutional trust while reducing uncertainty that taxes trauma-compromised working memory [11,12]. Guideline 3 (Self-Pacing Mechanisms): save-as-draft functionality, visible progress indicators, and explicit pause points that validate the user’s need to step away. It counters learned helplessness and the PTSD-related executive-function deficits, such as planning, sustained attention, and working memory, which cause rigid, multistep workflows to fail for trauma-affected users [11,12]. Guideline 4 (Institutional Trust Signals): prominent display of recognized organizational affiliations, government endorsements, and security certifications. It responds to trauma-eroded institutional trust, for which conventional credibility signals are often insufficient [12]. Guideline 5 (Context-Aware Support): curated resources tailored to user characteristics, including age-specific guidance, jurisdiction-relevant legal information, and culturally appropriate referrals. To operationalize the cultural, historical, and gender issues principle, this guideline further specifies gender- and culturally responsive content, which includes language that explicitly counters victim-blaming, a concern especially salient in the Korean sociocultural context. For instance, displaying a message such as “What do you do if someone is threatening to share your intimate images?” on the main page could potentially place the burden on victims who are already navigating a difficult reporting process. This framing may inadvertently suggest that resolving the situation falls primarily on the victim, which might feel less validating for those seeking support. To avoid this, the guideline mandates that the guidance never implicitly attributes responsibility to the victim-survivor [2,6]. Guideline 6 (Grounding Tools): accessible helpline links, supportive messaging during high-anxiety interaction points, and mobile-first crisis design reflecting the platforms through which victim-survivors most commonly seek help [5]. Consistent with the Peer Support principle, it also offers optional connection to vetted survivor and peer-support networks to address the isolation that frequently accompanies NCII victimization [42]; its grounding components target acute hyperarousal that can exceed a user’s window of tolerance at high-distress steps [12,13]. To prevent specific identification of the platform, visual interface details are omitted; instead, the final columns of Table 3 summarize the candidate design responses used as phase 2 evaluation stimuli.

### Phase 2: Domain-Informed Evaluation Results

#### Overview

CQR analysis yielded 5 thematic domains and 14 categories (Table 4). Following CQR conventions, frequency labels are used descriptively to indicate how many participants endorsed a category within this specific evaluator sample. Given the small and homogeneous sample, “General” should not be interpreted as population-level prevalence or generalizability. Rather, it indicates that a theme was consistently observed across the 8 domain-informed student evaluators in this preliminary study.

**Table 4.** CQR^a^ results: thematic domains and categories^b^.

Thematic domain and category	Core idea	Frequency	Representative quote
Initial perceptions			
Unclear site purpose	Users could not discern site purpose	General	“I had no idea what this site was for.”
Visual distrust	Design felt untrustworthy	Typical	“The color scheme made it feel cheap.”
Cognitive overload	Excessive information caused confusion	Typical	“So much information, I didn’t know what to click.”
Persona credibility			
Psychological plausibility	Trauma-informed behavior felt realistic	General	“Of course they’d want to avoid repeating steps.”
Behavioral journey fit	Reactions matched experience	Typical	“Wanting to double-check safety—that resonated.”
Need for diversity	More diverse personas needed	Variant	“Maybe add a male victim persona.”
UX^c^ feedback			
Trust-building features	Logos created confidence	General	“The official logo made me trust it more.”
Comforting elements	Language supported ease	Typical	“‘Find support’ felt much less intimidating.”
Improved flow	Save feature helped	Typical	“Saving progress made a huge difference.”
Emotional safety			
Trust based on prior knowledge	Trust needed context	General	“Without background info, I wouldn’t trust it.”
Fear of fake sites	Impersonation fears	Typical	“I’d question if this was real.”
Institutional endorsement	Official affiliation needed	Typical	“Endorsed by a government agency.”
Systemic barriers			
Search invisibility	Not found in search	General	“I searched and it doesn’t show up.”
Desire for human support	Real-time help wanted	Variant	“A chat option mid-process would help.”

a CQR: consensual qualitative research.

b General=7-8 of 8 participants, typical=4-6, and variant=2-3.

c UX: user experience.

#### Domain 1: Initial Perceptions

The platform’s purpose was not readily apparent to participants (general). Aesthetic elements, particularly color choices and layout density, elicited active distrust rather than merely failing to inspire confidence (typical). Information density created cognitive paralysis, with participants reporting inability to identify relevant next steps (typical).

#### Domain 2: Persona Credibility

Personas were received as psychologically plausible, particularly regarding trauma-shaped behaviors: repeated safety checking before commitment, hesitation at irreversible steps, and need for institutional legitimacy verification (general). Journey narratives were emotionally resonant (typical), with participants reporting that they could envision users exhibiting the personas’ described hesitations and retreat patterns. The variant-level concern about exclusively female representation warrants attention in future iterations. General-level endorsement provides preliminary support for the plausibility of the diagnostic methodology within this evaluator group, but it does not establish ecological validity with NCII victim-survivors. This finding partially addresses the methodological concern that personas constructed without direct user research risk stereotyping [28,30], while also underscoring the need for subsequent survivor-centered evaluation. Furthermore, domain-informed plausibility rated by participants trained in the same field presents a lower bar than it might appear; establishing external validity ultimately requires evaluators outside this disciplinary overlap.

#### Domain 3: UX Design Feedback

Institutional logos transformed perceptions from “potentially fraudulent” to “officially endorsed,” with participants describing a categorical rather than incremental shift in willingness to engage (general), supporting guideline 4. Language changes that replaced “Create a case” with “Find support” were perceived as substantially more comforting and less bureaucratic (typical), supporting guideline 1. The save-as-draft function received positive responses (typical), with multiple participants noting that the original rigid workflow would be prohibitive during emotional distress, providing preliminary support for the clinical rationale underlying guideline 3. This convergence between clinical prediction and evaluator feedback suggests that mental health expertise may improve early-stage safety design, although behavioral effectiveness remains untested.

#### Domain 4: Emotional Safety

Trust was conditional on external endorsement: participants required a trusted intermediary (counselor, teacher, and government website) to vouch for the platform before engaging, regardless of interface quality (general). Fraudulent website fears persisted even after viewing framework-informed interface examples with improved trust signals (typical).

#### Domain 5: Systemic Barriers

Search engine invisibility was the most prominent systemic concern (general): participants could not locate the platform through standard search queries, rendering interface improvements irrelevant for users who never arrive. Desire for real-time human support was noted (variant), consistent with TIC’s Collaboration and Mutuality principle. These findings identify structural prerequisites beyond interface design, including search engine optimization strategy, institutional referral partnerships, and integrated support service connections, consistent with the argument by Chen et al [1] that trauma-informed technology requires systemic organizational transformation, not merely interface-level changes, and with Safety by Design’s Holistic principle requiring safety considerations across the full product life cycle [16].

## Discussion

### Principal Findings

This study developed and preliminarily evaluated a TID framework for NCII removal platforms. Applying trauma-differentiated personas to an existing platform (RQ1) surfaced 5 crosscutting barriers, including ambiguous eligibility language, emotionally triggering content, absent emotional support, opaque data handling, and low institutional credibility, which conventional usability evaluation alone may not fully capture. Jointly operationalizing TIC, Safety by Design, and usability heuristics (RQ2) produced 6 TID guidelines. In domain-informed evaluation (RQ3), evaluators judged the personas plausible and viewed design responses featuring institutional trust signals, self-pacing, and supportive language as emotionally safer and potentially more usable, while emphasizing that survivor-centered validation remains necessary. The study’s primary contribution is to operationalize the Safety by Design mandate for NCII removal platforms: pairing TIC, as clinical content, with usability heuristics, as interaction mechanics, to turn a broad protective obligation into concrete, actionable design guidance, thereby helping to close the gap between safety policy frameworks and interface-level guidance.

### TIC as a Foundational Framework for Digital Safety Design

The central finding is that the barriers that arise on NCII removal platforms are rooted in the psychological consequences of trauma, and that TIC provides the theoretical apparatus necessary to diagnose them. The rigid, save-progress-free workflow in Rina’s journey map illustrates this: in usability terms a minor violation of Nielsen’s [9] user-control heuristic, but through a TIC lens a reproduction of learned helplessness, as effort expended and then arbitrarily erased mirrors the powerlessness Herman [12] identified as central to prolonged interpersonal trauma, inadvertently replicating how abusers exploit technology to maintain control [23]. Likewise, ambiguous data-handling policies (a transparency gap in usability terms) activate hypervigilant threat monitoring in users whose institutional trust is compromised [3], and information-dense interfaces (an aesthetic-minimalism violation) exceed the diminished executive function capacity documented in PTSD [11]. This pattern (the same feature receiving qualitatively different diagnoses depending on whether TIC principles are applied) illustrates why TIC is foundational to this diagnostic approach. Prior work mapped heuristic-TIC convergences, demonstrating that the 2 traditions share substantial common ground [14].

Consistent with this interpretation, evaluators’ difficulty discerning the platform’s purpose and their distrust of information-dense screens map onto the trauma-compromised attention, working memory, and executive function documented in PTSD populations [11], such that information-dense interfaces function as inaccessible rather than merely suboptimal environments. Likewise, the conditioning of trust on external endorsement reflects trauma-eroded institutional trust [12]: for trauma-affected users, procedural transparency is necessary but insufficient without validation from trusted institutions, implying that referral partnerships with counseling services, government agencies, and NGOs may matter more for initial engagement than interface improvements alone.

### A Diagnostic Methodology Bridging 3 Traditions

The combination of trauma-differentiated personas with TIC-principled journey mapping provides a methodology that may surface concerns invisible to any single tradition. General-level persona endorsement provides preliminary support for the approach within this domain-informed student evaluator group and demonstrates the unreliability of persona construction without clinical grounding [30]. Centering victim-survivor experiences remains important for digital tool development in the gender-based violence context [2]. The present work does not substitute for such survivor involvement; our contribution is complementary: TID provides a diagnostic method for an earlier phase when direct survivor involvement may not yet be ethically appropriate, producing evidence-grounded foundations that subsequent survivor-centered evaluation can refine and validate. The persona methodology serves as the integration mechanism, whereby personas embody clinical trauma profiles [12,13] in a format that UX practitioners can apply through journey mapping and that safety engineers can evaluate against risk frameworks [16]. Because clinicians, UX practitioners, and safety engineers each work with diagnostic tools the others lack, surfacing the full range of barriers requires integrating the 3 perspectives rather than relying on any single tradition.

### Implications for Policy and Platform Design

These findings also reframe the purpose of TID. Its value lies less in serving as an ethics checklist or a compliance layer added to an existing system than in functioning as psychologically realistic interaction design that anticipates the predictable states of a help-seeking population (reduced control, avoidance, dissociation, and the anticipated cost of disclosure or refusal) and specifies how an interface should respond when a user’s capacity to negotiate, object, or reengage is compromised. On this view, the features evaluators endorsed most strongly (institutional trust signals, self-pacing mechanisms, and grounding supports) function not as courtesies but as the system’s relational response, situating TID within the interaction model rather than in supplementary policy. This framing extends the logic of survivor-centered conversational systems [43,44] from stand-alone agents to the removal workflow itself.

TID also continues a cumulative design lineage (from Privacy and Security by Design to Safety by Design) that progressively widens the categories of user vulnerability a system is expected to anticipate. As the Digital Services Act [45] and Online Safety Act [46] translate Safety by Design into regulatory obligation, TID offers a candidate interaction-level method for rendering those obligations as user-facing criteria, although transferability should be claimed cautiously. Building on calls for empirically evaluable trauma-informed frameworks [5], we propose a standardized TID heuristic spanning interface-level criteria (emotional tone, cognitive load, and pacing) and systemic criteria (referral pathways and institutional partnerships), applied alongside conventional heuristic evaluation and with clinical expertise embedded in requirements definition rather than added as post hoc consultation.

### Limitations and Future Directions

Several limitations warrant acknowledgment. First, the framework was not evaluated with NCII victim-survivors or with users actively seeking image-removal support. This decision was ethically motivated, given the sensitivity of NCII, the possibility of retraumatization, and the need to establish preliminary procedural safety before involving survivors. However, it constrains the ecological validity of the present findings. Future research should evaluate the framework with NCII victim-survivors through ethically robust, survivor-centered methods [2,47]. Second, phase 2 involved a single focus group with 8 female Counseling-UX Psychology university student evaluators. CQR frequency labels provide useful within-sample descriptors, but they do not establish saturation across populations or prevalence estimates. The small, homogeneous, domain-informed student sample may respond differently from victim-survivors, users without counseling-UX training, users with lower digital literacy, or users in acute crisis. Third, all personas and participants were female. Although this reflects the strongly gendered distribution of NCII abuse [6], in nationwide data, women are nearly twice as likely as men to be targeted by nonconsensual intimate imagery [48], and the overwhelming majority of sexual deepfakes is nonconsensual and almost exclusively depicts women [49]. Nonetheless, the variant-level diversity concern raised by participants underscores the need to broaden future persona sets across gender, sexuality, age, disability status, and cultural contexts. Fourth, the framework was developed through a single NCII-focused case and should not be treated as fully transferable to all trauma-related digital services; some findings are likely NCII-specific (eg, fear of image circulation and shame around uploading intimate material), whereas elements such as procedural transparency, user-controlled pacing, and grounding support may transfer to adjacent digital safety contexts. Finally, the dual-lens method is, by design, theory-driven: it applies TIC as an analytic lens to surface barriers that usability evaluation alone tends to leave unexamined. Its contribution is accordingly generative (identifying and specifying candidate barriers and design responses) rather than confirmatory, and establishing that a trauma-informed lens yields outcomes superior to conventional evaluation would require controlled comparative testing, which we identify as a priority for subsequent work.

### Conclusions

Emotional safety is a design requirement, not an enhancement: for platforms meant to help people already harmed, designing for the user who cannot readily advocate for themselves is what separates a tool that protects from one that may reproduce the original loss of control. Jointly operationalized through a clinically informed, persona-based diagnostic method, TIC, Safety by Design, and usability principles surfaced psychological gaps that no single method detects alone; the 6 guidelines are an actionable but preliminary starting point, and the personas and journey maps show how Safety by Design can be applied concretely rather than as aspiration. The framework now requires survivor-centered, cross-cultural, and behavioral evaluation before claims of effectiveness or broad transferability. Embedding these principles in removal platforms may enhance both their usability and their adoption, increasing the likelihood that the support these systems are designed to provide reaches the victim-survivors who seek it.

## Supplementary material

10.2196/94438 Multimedia Appendix 1 Focus group guide.

## Abbreviations

CQR: consensual qualitative research
NCII: nonconsensual intimate image
PTSD: posttraumatic stress disorder
RQ: research question
TIC: trauma-informed care
TID: trauma-informed design
UX: user experience
