# Supplementary material for: Designing a Trauma-Informed Safety by Design Framework for Nonconsensual Intimate Image Removal Platforms: A 2-Phase Sequential Exploratory Study
Source: JMIR Hum Factors. 2026 Aug 10;13:e94438. doi: 10.2196/94438 (PMC13455849; doi:10.2196/94438)
Supplement: Supplementary file 1 [file humanfactors-v13-e94438-s001.docx]

**Multimedia Appendix 1**

**Focus group guide**

The 1-hour semi-structured focus group followed the structure below (times in minutes). The guide was used flexibly to allow emergent discussion while maintaining comparability across sections.

Before block 3, participants were given a brief explanation of the TID framework: six design guidelines—emotional scaffolding, process transparency, self-pacing mechanisms, institutional trust signals, context-aware support, and grounding tools—derived from trauma-informed care and usability heuristics under Safety by Design, which the TID-informed interface examples instantiate.

| **Section and questions** | **Facilitator notes** | **Min** |
| --- | --- | --- |
| Introduction and ground rules. Introduce the research objectives, the research team, and the focus-group procedure. Emphasize that the session evaluates platform design, the personas, and the TID-informed interface examples, not participants’ personal NCII or trauma experiences. Explain voluntary participation, confidentiality, the possibility of discomfort, and the right to skip any question or withdraw without penalty, and confirm that written informed consent has been obtained. | Distribute informed-consent forms; remind participants not to disclose personally identifying or traumatic details. | 5 |
| Introductory question. (1) When you hear “NCII” or “NCII dissemination” in the context of platform safety, what user needs, risks, or safeguards come to mind? |  | 5 |
| Transition question. (2) When you first reviewed this NCII removal platform’s interface, what stood out to you—positively or negatively? |  | 5 |
| Persona plausibility. (3) Do these personas seem plausible and internally coherent, or do any parts feel forced, and what makes them feel more—or less—realistic? (4) How well does each persona’s platform behavior fit their profile, and where does it feel matched or mismatched? (5) Drawing on your coursework and training in counseling psychology and UX, how plausible are these personas’ emotional reactions, and are any over- or understated? (6) Do these 3 personas adequately represent the range of users this platform may serve, or are perspectives left out? | Distribute the persona summaries and user-journey maps. | 15 |
| Comparison of original and TID-informed interface examples. (7) Starting with the landing page, comparing the TID-informed interface example with the original, what differences do you notice in how a user might experience it, and which elements would increase or decrease a user’s sense of safety? (8) Moving to the case-creation section, what stood out to you, positively or negatively, and why? (9) In the platform-introduction and eligibility sections, how clear are the guidance and eligibility criteria, and how might they affect a user in distress? (10) Overall, how would the TID-informed interface example affect how secure or comfortable each persona feels compared with the original (please specify the persona and the design element)? (11) Are there aspects of the TID-informed interface example that a user might find more difficult, confusing, or distressing than the original? (12) Thinking about the six TID guidelines just described, do the TID-informed changes follow sensibly from them, and does the framework make sense as a basis for supporting trauma-affected users on this platform? | Distribute reference images comparing the original interface with the TID-informed interface examples. | 15 |
| Willingness to use. (13) Hypothetically, if someone in your social circle experienced an NCII-related incident, would you consider recommending this platform, and which features would increase or reduce that likelihood? | Emphasize that this is hypothetical; participants may decline if uncomfortable. | 5 |
| Ending question. (14) Is there anything important about persona plausibility, emotional safety, or platform design that we should have discussed but did not? |  | 5 |
| Total |  | 60 |

Note. The TID-informed interface examples used in block 3 were low-fidelity instantiations of the 6 guidelines (Table 3), prepared to support side-by-side comparison and evaluation rather than as finalized designs for any platform.
